# Supplementary material for: miR-542-3p Contributes to the HK2-Mediated High Glycolytic Phenotype in Human Glioma Cells
Source: Genes (Basel). 2021 Apr 23;12(5):633. doi: 10.3390/genes12050633 (PMC8146800; doi:10.3390/genes12050633)
Supplement: Supplementary file 1 [file genes-12-00633-s001.zip › genes-1195893-supplementary.pdf]

Supplemental data

miR-542-3p contributes to HK2-mediated high glycolytic phenotype in human glioma cells.

Junhyung Kim [1#], Min Woo Park [1#], Young Joon Park [2], Ju Won Ahn [2], Jeong Min Sim [2], Suwan Kim [2], Jinhyung Heo [3], Ji Hun Jeong [1], Mihye Lee [1], Jaejoon Lim [2*], and Jong-Seok Moon [1*]

Supplemental Table 1

Supplemental table 1 Information for patients with glioma

Patient	Diagnosis	Grade	Age	Sex	Ki-67	IDH mutation
P1	ganglioglioma	I	28	M	1%	Wild-type
P2	ganglioglioma	I	45	M	5%	Wild-type
P3	oligodendroglioma	II	40	M	5%	Mutation
P4	diffuse astrocytoma	II	33	M	5%	Wild-type
P5	diffuse astrocytoma	II	75	F	7%	Wild-type
P6	anaplastic astrocytoma	III	34	M	5%	Wild-type
P7	anaplastic astrocytoma	III	56	F	20%	Wild-type
P8	anaplastic oligodendroglioma	III	46	M	10%	Mutation
P9	glioblastoma	IV	37	F	20%	Wild-type
P10	glioblastoma	IV	70	F	30%	Wild-type
P11	glioblastoma	IV	59	F	10%	Wild-type

+; Alive, M; Male, F; Female
